# Effect of Herbal Prescriptions in Accordance with Pattern Identification in Acute Cerebral Infarction Patients: Based on Fire-Heat Pattern

**DOI:** 10.1155/2015/517158

**Published:** 2015-10-07

**Authors:** WooSang Jung, JungMi Park, SangKwan Moon, Sangho Hyun

**Affiliations:** Department of Cardiology and Neurology of Korean Medicine, College of Korean Medicine, Kyung Hee University, Hoegi-dong, Dongdaemun-gu, Seoul 130-702, Republic of Korea

## Abstract

*Objectives.* This study was conducted to verify the necessity of corresponding prescription to the diagnosed pattern in acute cerebral infarction patients. *Methods.* We studied cerebral infarction patients hospitalized within 30 days after the ictus. Forty-four clinical indicators, Motricity Index (MI) score, Scandinavian Stroke Scale (SSS) score, and herbal prescriptions were checked twice, two weeks apart. The probability of each pattern was calculated based on the clinical indicators. Changes in MI score, SSS score, and the probability of fire-heat pattern were compared between the pattern-prescription correspondence group and the noncorrespondence group. *Results.* Increments of MI score and SSS score in the correspondence group were significantly greater than those of the noncorrespondence group (*p* = 0.003, *p* = 0.001) while the baseline score of the two groups showed no significant difference. Probability of fire-heat pattern decreased significantly in the correspondence group (*p* = 0.013) while the noncorrespondence group showed no significant difference after the treatment. *Conclusion.* Acute cerebral infarction patients who are diagnosed as fire-heat pattern showed better improvement in dysfunctions caused by the disease when they took the pattern corresponding prescriptions. This study provides evidence for the necessity and usefulness of pattern identification in Traditional Korean Medicine.

## 1. Introduction

In South Korea, the political and social status of Traditional Korean Medicine (TKM) almost equals that of Western medicine. This is due to a strong preference for TKM in Korean people. When 1,000 people living in Seoul were asked to choose between Western medicine doctors and TKM doctors if they had developed a stroke, 25% of the subjects chose TKM doctors, and 45% of the subjects responded that they were willing to receive TKM treatments [1]. Also, a survey demonstrated that 40% of stroke patients tried TKM treatments after they were discharged from a Western medicine hospital [2]. Among many diseases, TKM has been favored especially for the treatment of stroke.

Considering the large role in the treatment of stroke, significant amount of effort was put into accumulating scientific evidence of the efficacy and safety of TKM. As a result, questionnaire for the pattern identification and guidelines for assessing the clinical indicators were developed over the years [3, 4]. These guidelines allowed us to accumulate coherent data, and by using these data, we were able to work out equations for standardization of pattern identification for stroke patients [5, 6]. These works enhanced the objectivity and reproducibility of pattern identification and remedied its shortcomings. However, the necessity and usefulness of pattern identification have not been verified yet. Many herbal prescriptions have been proven to have a beneficial effect in acute stroke patients [7–9], but the relation between the efficacy and the pattern identification was not indicated.

In this study, to verify the necessity and usefulness of pattern identification, we compared the two groups of the patients diagnosed as fire-heat pattern. The group who took the herbal prescriptions in accordance with the pattern identification and the group of people who did not take the herbal prescriptions accordingly were compared to demonstrate if the corresponding prescription taking group shows better outcome. Also, we performed a correlation study between the changes in clinical indicators and the improvement in dysfunctions to identify if the changes in symptoms are relevant to recovery of poststroke dysfunctions.

## 2. Materials and Methods

### 2.1. Subjects

We enrolled ischemic stroke patients within 30 days after their ictus from Kyung Hee Korean Medical Center and Kyung Hee East-West Neo Medical Center. Imaging diagnosis such as computerized tomography (CT) or magnetic resonance imaging (MRI) was checked to confirm the ischemic stroke. We excluded traumatic strokes such as subarachnoid, subdural, and epidural hemorrhage. Also, we excluded patients with brain tumor, Alzheimer's disease, multiple sclerosis, or any other neurodegenerative diseases. Informed consent of all the participants was obtained after a thorough explanation of the details. Over a 3-year period from May 2011 to January 2014, 300 patients were included in the study. The Institutional Review Board of the Kyung Hee Korean Medical Center and Kyung Hee East-West Neo Medical Center approved the present study (KOMCIRB-2011-02, KOMCIRB-2012-04, KHNMCOHIRB-2011-002, and KHNMCOHIRB-2012-003).

### 2.2. Study Design and Interventions

After the admission, two different TKM doctors identified the pattern of each patient based on the clinical indicators they show, and we confirmed the pattern only if the two TKM doctors had the same opinion. We used the Case Report Form (CRF) and the Standard Operation Procedures (SOP) developed by the Korean Institute of Oriental Medicine [3, 10] to reduce inconsistency in pattern identification carried out by different TKM doctors.  Table 1 shows the forty-four clinical indicators contained in the CRF, and there were four possible patterns to choose from, which were fire-heat pattern, Yin Deficiency Pattern, Phlegm Dampness Pattern, and Qi Deficiency Pattern. The patients whose patterns were not decided because the opinions of the two TKM doctors differed were dropped out. After the pattern identification, the patients were allocated into each group according to their confirmed pattern.

All subjects were studied twice, 2 weeks apart. During the 2-week period, all participants received conventional Western medicine treatment such as antiplatelet agent, risk factor control (e.g., hypertension, diabetes mellitus, dyslipidemia, and cardiac disease), and rehabilitation exercise. TKM treatment was also administered to all of the patients, which includes herbal prescription, acupuncture, and electroacupuncture. The contents of acupuncture and electroacupuncture treatment are shown in Table 2. The herbal prescriptions applied to each patient were selected according to the patient's condition and associated symptoms by the TKM doctors who were irrelevant to the present study. The prescriptions used during the treatment period were checked, and we classified the prescriptions based on the guideline suggested in the SOP (Table 3).

### 2.3. Measurements

Baseline characteristics such as age, sex, Body Mass Index (BMI), period from onset to admission, medical history, alcohol and smoking habits, and Trial of Org 10172 in Acute Stroke Treatment (TOAST) classification [11, 12] of the stroke types were checked. To estimate the motor function, we used Motricity Index (MI) score [13], which is a reliable scale in assessing motor impairment after stroke. Scandinavian Stroke Scale (SSS) score [14] was used to evaluate the degree of dysfunctions in the subjects. The assessors for the MI and SSS scores did not have the information about the herbal prescriptions the patients are taking.

To assess the changes in the clinical indicators, we used the logistic equations for calculating the probability of each pattern suggested by Kim et al. [5]. The same CRF and SOP used in the present study were used in their research, and the logistic equations were derived based on the clinical data of 480 stroke patients as a result of regression analysis. The equations for the probability of four patterns are as follows:

(1) A=3.021×reddened  complexion+1.052×eyeball  congestion+0.682×aversion  to  heat−1.388pale  tongue+0.727×thick  fur−1.134×teeth  marked  tongue+1.295×strong  pulse−1.122×thin  pulse−0.972×slippery  pulse−2.865,B=3.552×flushed  cheeks+1.024×thirst+1.740×afternoon  tidal  fever+0.963×dry  fur+0.982×rapid  pulse−1.932×strong  pulse−3.705,C=0.578×overweight−0.754×fatigue−1.754×pale  complexion−2.189×reddened  complexion−2.719×flushed  cheeks+1.496×pale  tongue+2.365×slippery  pulse−1.136,D=−0.882×overweight+2.417×pale  complexion−2.869×reddened  complexion−2.252×flushed  cheeks+1.451×eyeball  dryness−1.577×night  sweating−1.474×nausea+1.165×reversal  cold  of  the  extremities−2.100×thick  fur+0.783×deep  pulse−2.214×rapid  pulse+0.993×vacuous  pulse−2.572×slippery  pulse−0.907.

Put in “1” for the existing clinical indicators and “0” for the nonexisting clinical indicators.

*Probability of Fire-Heat Pattern*. Consider

(2) $$\begin{array}{c} P_{FHP}=\frac{e^{A}}{\left(1+e^{A}\right)}. \end{array}$$

*Probability of Yin Deficiency Pattern*. Consider

(3) $$\begin{array}{c} P_{YDP}=\frac{e^{B}}{\left(1+e^{B}\right)}. \end{array}$$

*Probability of Phlegm Dampness Pattern*. Consider

(4) $$\begin{array}{c} P_{PDP}=\frac{e^{C}}{\left(1+e^{C}\right)}. \end{array}$$

*Probability of Qi Deficiency Pattern*. Consider

(5) $$\begin{array}{c} P_{QDP}=\frac{e^{D}}{\left(1+e^{D}\right)}. \end{array}$$

Patients that display more fire-heat pattern related symptoms show higher probability of fire-heat pattern. The discriminant validity of the equations for the probability of the four patterns is shown in Table 4.

### 2.4. Statistical Analysis

Statistical analysis was performed by using the Statistical Package for the Social Sciences version 12.0 for Windows (SPSS, Chicago, IL). Chi-square test was used for the categorical variables, and Mann-Whitney test was used for the continuous variables when comparing the two groups. Wilcoxon signed rank test was used for statistical comparisons between the values before and after the treatment. We correlated the changes in the probability of fire-heat pattern with the changes of SSS score and MI score, respectively, using Spearman's rank correlation. A *p* < 0.05 was considered significant.

## 3. Results

**Figure 1:**
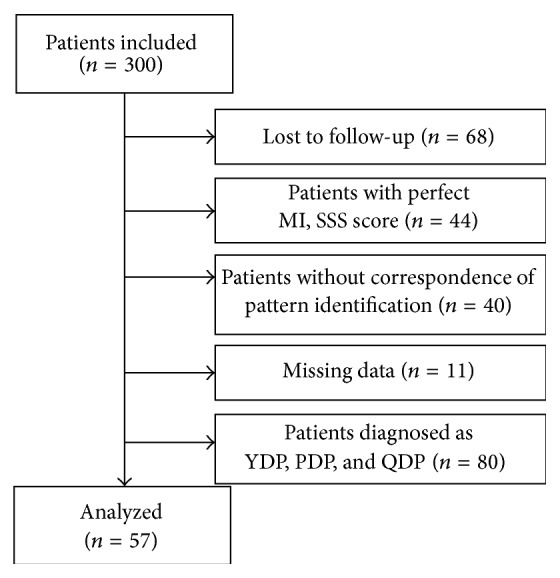
Flowchart of patients enrolled in this study. MI, Motricity Index; SSS, Scandinavian Stroke Scale; YDP, Yin Deficiency Pattern; PDP, Phlegm Dampness Pattern; QDP, Qi Deficiency Pattern.

Of the 300 patients enrolled in the study, 68 patients were discharged before the second checkup, and 44 patients with perfect MI and SSS score were excluded as they could not expect further improvement. 40 patients were unable to determine the pattern because the diagnosis of the two TKM doctors differed. 11 patients were dropped out due to missing data. Also, many patients diagnosed as Yin Deficiency Pattern, Phlegm Dampness Pattern, and Qi Deficiency Pattern received different types of herbal prescriptions during the treatment period. Only two patients in the Yin Deficiency Pattern, three patients in the Phlegm Dampness Pattern, and two patients in the Qi Deficiency Pattern received pattern corresponding herbal prescription for the whole 2-week period, so we were unable to secure a sufficient sample size for statistical analysis for those three patterns. Among the remaining 57 patients who were diagnosed as fire-heat pattern, we considered 40 patients who received herbal prescriptions targeting fire-heat pattern related symptoms into correspondence group and the other 17 patients who received herbal prescriptions focusing on clinical indicators of other patterns into noncorrespondence group (Figure 1). After 2-week period of treatment, no aggravation of the neurologic deficit was observed in the patients.

### 3.1. Baseline Assessment

General characteristics, period from onset to admission, medical history, alcohol and smoking experience, and proportion of ischemic stroke type according to TOAST classification showed no significant difference between the two groups (Table 5).

### 3.2. MI Score and the SSS Score before and after the Treatment

Both groups showed increase in the MI score and the SSS score, but the increments of the MI score and the SSS score in the correspondence group were significantly greater than those of the noncorrespondence group (*p* = 0.003, *p* = 0.001) while the baseline scores of the two groups showed no significant difference (Table 6).

### 3.3. Changes in the Probability of Fire-Heat Pattern

The probability of fire-heat pattern was significantly higher than the probability of other patterns in both groups (*p* < 0.0001). The baseline probability of fire-heat pattern between the two groups showed no significant difference. The probability of fire-heat pattern decreased significantly after the treatment in the correspondence group (*p* = 0.013) while the probability of fire-heat pattern in noncorrespondence group showed no significant change. Probability of other patterns showed no significant change after the treatment in both groups (Table 7).

### 3.4. Correlation Analysis in the Fire-Heat Pattern Corresponding Prescription Group

**Figure 2:**
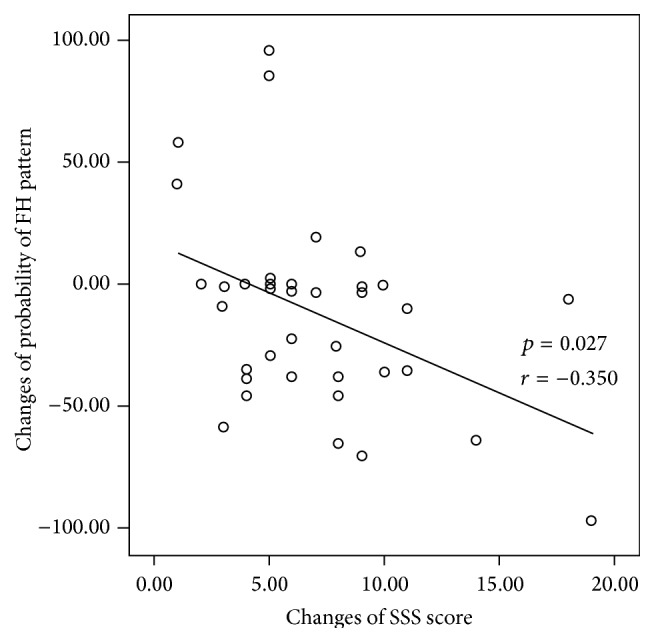
Correlation analysis between the changes of SSS score and the probability of fire-heat pattern in the correspondence group (*p* = 0.027, *r* = −0.349).

**Figure 3:**
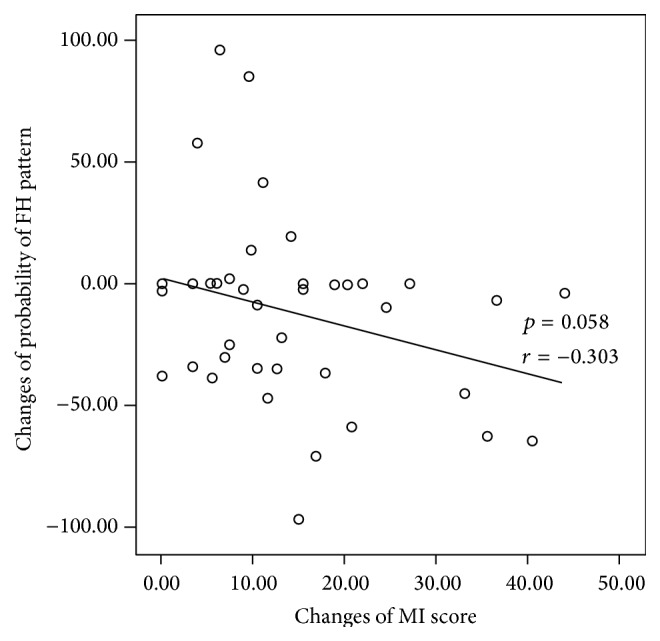
Correlation analysis between the changes of MI score and the probability of fire-heat pattern in the correspondence group (*p* = 0.058, *r* = −0.303).

In the correlation study, the decrease in the probability of fire-heat pattern showed significant correlation with the increase in the SSS score (*p* = 0.027) and missed statistical significance with the increase in the MI score (*p* = 0.058) (Figures 2 and 3).

## 4. Discussion

The aim of this study was to verify the usefulness of the pattern identification. To achieve this goal, we compared the outcome of the treatments in pattern-prescription correspondence group and the noncorrespondence group. While the baseline scores did not differ significantly between the two groups, increments of MI score and SSS score after the treatment were significantly higher in the correspondence group than the noncorrespondence group (*p* = 0.003, *p* = 0.001). This suggests that taking herbal prescriptions in accordance with the diagnosed pattern is more effective in improving functional impairments of acute ischemic stroke patients diagnosed as fire-heat pattern.

A type of herbal prescription is selected based on the clinical indicators a patient is showing, and when used, the herbal prescriptions are expected to alleviate the clinical symptoms. We used the probability of fire-heat pattern as a scale to evaluate the changes in the clinical symptoms of patients diagnosed as fire-heat pattern. As expected, the probability of fire-heat pattern decreased significantly in the correspondence group (*p* = 0.013) while there were no significant changes in the noncorrespondence group. Also, correlation study indicates that the patients with larger increment in the SSS score showed larger decrement in the probability of fire-heat pattern (*p* = 0.027). In our previous study concerning the treatment of acute ischemic stroke patients, motor function recovery in the patients correlated significantly with the improvement in the symptoms related to fire-heat pattern [15], which is consistent with the results of the present study. These results suggest that patients with improved functional impairments tend to show alleviation of clinical symptoms related to fire-heat pattern.

Usage of herbal prescriptions on acute ischemic stroke patients has been studied over the years [7–9], but no research was carried out to verify the necessity and usefulness of the pattern identification. Pattern identification is a meaningful diagnostic tool of TKM as it allows individualized treatment, maximizing its effectiveness and minimizing its adverse effects. We tried comparing the pattern-prescription corresponding group and noncorresponding group in our study in 2011, but the sample size was too small and the treatment period was too short, and the results showed no statistical significance [16]. This is the first study to evaluate the effectiveness of pattern identification in acute ischemic stroke patients diagnosed as fire-heat pattern.

We could not verify the effectiveness of pattern identification in patients diagnosed as Yin Deficiency Pattern, Phlegm Dampness Pattern, and Qi Deficiency Pattern, because they were not consistent in consuming the herbal prescriptions corresponding with their pattern and therefore were not suitable for the subjects of this study. This was not expected when we designed the study, but due to this outcome, the application of the results in the present study should be limited only in the patients diagnosed as fire-heat pattern. Another limitation is that the probability of pattern does not properly evaluate the severity of the clinical symptoms related to each pattern since the scale was made to determine the pattern not to assess the clinical indicators. We used the probability of patterns in this study because this scale was created based on the data collected using the same CRF and SOP used in the present study. There is no widely accepted scale assessing the severity of the symptoms related to pattern identification, and it should be developed in the future for more researches concerning pattern identification.

In the present study, correspondence group displayed better outcome than the noncorrespondence group, and in the correspondence group, patients with lesser clinical indicators related to fire-heat pattern after the treatment showed better improvement in the recovery of functional impairment. These results imply that herbal prescriptions in accordance with the diagnosed pattern alleviate the clinical symptoms in relation with the diagnosed pattern and are more effective in restoring the dysfunctions caused by the disease than the noncorresponding prescriptions. The results provide evidence for the necessity and usefulness of pattern identification in TKM, but further research is needed to confirm the effectiveness of pattern identification for the other pattern groups.

## Figures and Tables

**Table 1:** Clinical indicators related to pattern identification.

Overweight	Body Mass Index >23 (kg/m^2^)
Insomnia	Inability to sleep or abnormal wakefulness
Fatigue	Lack of strength
Pale complexion	A white complexion with a hint of blue or gray, often caused by yang collapse or exuberance of cold
Yellow complexion	Yellow discoloration of the face, generally suggesting accumulation of dampness
Reddened complexion	A complexion redder than normal, indicating the presence of heat
Darkish complexion	Dark discoloration of the face, often occurring in cold syndrome, water retention, or blood stasis
Flushed cheeks	Localized flush in the cheeks, indicating yin deficiency
Headache	Pain in the head
Eye congestion	Congestion in eyeballs indicating presence of heat
Eyeball dryness	Subjective feeling of dryness in the eyeballs
Phlegm rale	An abnormal breathing sound by phlegm in the airways
Faint low voice	A voice that is faint and low, scarcely audible
Tongue sore	Ulceration in the oral cavity or tongue
Halitosis	Bad smell from the mouth
Thirst	Feeling of dryness of the mouth with a desire to drink
Bitter taste in the mouth	A subjective bitter sensation in the mouth
Night sweating	Sweating during sleep that ceases on awakening
Chest discomfort	Unwell feeling of stuffiness and fullness in the chest
Nausea	An unpleasant sensation with an urge to vomit
Aversion to heat	Strong dislike of heat, also known as heat intolerance
Afternoon tidal fever	Fever more marked in the afternoon
Heat in the palms and soles	Subjective feverish feeling in the palms and soles
Vexing heat in the extremities	Uncomfortable heat sensation in the extremities
Reversal cold of the extremities	Pronounced cold in the extremities up to the knees and elbows, also the same as cold extremities
Reddish yellow urine	Dark yellow or even reddish urine, indicating heat
Pale tongue	A tongue less red than normal, indicating Qi and blood deficiency
Red tongue	A tongue redder than normal, indicating the presence of heat
White fur	A tongue coating white in color
Yellow fur	A tongue coating yellow in color
Thick fur	A tongue coating where the underlying tongue surface is not visible
Dry fur	A tongue coating that looks dry and feels dry to the touch
Teeth marked tongue	A tongue with dental indentations on its margin
Enlarged tongue	A tongue that is larger than normal, pale in color, and delicate
Mirror tongue	A completely smooth tongue free of coating, like a mirror
Floating pulse	A superficially located pulse which can be felt by light touch and grows faint on hard pressure
Deep pulse	A deeply located pulse which can only be felt when pressing hard
Slow pulse	Bradycardia
Rapid pulse	Tachycardia
Strong pulse	A general term for strongly beating pulse
Vacuous pulse	A general term for a feeble and void pulse
Thin pulse	A pulse as thin as a silk thread, straight and soft, and feeble yet always perceptible upon hard pressure
Slippery pulse	A pulse coming and going smoothly like beads rolling on a plate
Flooding pulse	A pulse beating like dashing waves with forceful rising and gradual decline

**Table 2:** Traditional Korean Medicine treatments applied in the study.

Treatment	Contents
Acupuncture (once a day)	LI4, LI11, ST36, LR3, GB20 (both sides), TE5, LI10, ST37, GB39, GB34, SP3, SP4 (debilitated side), GV20, GV26, and CV24
Electroacupuncture (once a day)	LI4, TE5, LI10, LI11, ST36, ST37, GB39, and LR3 (debilitated side)

**Table 3:** Classification of prescriptions used in this study by Korean Institute of Oriental Medicine.

Fire-heat pattern	Yin Deficiency Pattern	Phlegm Dampness Pattern	Qi Deficiency Pattern
Yangkyuksanwha-tang	Hyungbangjihwang-tang	Bosimgunbi-tang	Sunghyangjunggi-san
Chungpyesagan-tang	Dokhwaljihwang-tang	Banhabaekchulchunma-tang	Bojungikgi-tang
Yeoldahanso-tang	Jaumganghwa-tang	Sunkidodam-tang	Ssanghwa-tang
Chungsim-tang	Yukmijihwang-tang	Gami-ondam-tang	Boyanghwano-tang
Jihwangbakho-tang	Saryuk-tang		Yikgeebohyul-tang

**Table 4:** Discriminant validity of probability of four patterns.

	Probability of FHP	Probability of YDP	Probability of PDP	Probability of QDP	*p* value
FHP group (*n* = 57)	58.7 (38.8)	14.0 (32.0)	10.9 (25.7)	1.4 (8.6)	<0.0001
YDP group (*n* = 27)	17.8 (33.0)	31.2 (42.0)	16.5 (32.1)	4.8 (13.9)	<0.0001
PDP group (*n* = 30)	16.6 (33.9)	0.8 (2.3)	59.9 (42.7)	4.5 (16.2)	<0.0001
QDP group (*n* = 23)	0.1 (0.3)	4.2 (11.8)	19.6 (35.7)	38.2 (43.7)	<0.0001

FHP, fire-heat pattern; YDP, Yin Deficiency Pattern; PDP, Phlegm Dampness Pattern; QDP, Qi Deficiency Pattern.

**Table 5:** Comparisons of baseline characteristics between the correspondence group and the noncorrespondence group.

	Correspondence group (*n* = 40)	Noncorrespondence group (*n* = 17)	*p* value
Gender, male (%)	24 (60.0)	8 (47.1)	0.397
Age, yr (SD)	69.2 (10.0)	68.4 (10.0)	0.524
BMI, kg/m^2^ (SD)	24.1 (3.0)	24.5 (3.4)	0.848
Treatment period from onset, day (SD)	9.5 (6.2)	12.7 (8.7)	0.142
Past history			
Hypertension (%)	32 (80.0)	12 (70.6)	0.499
Dyslipidemia (%)	15 (37.5)	7 (41.2)	1.000
Diabetes mellitus (%)	15 (37.5)	6 (35.3)	1.000
Heart disease (%)	4 (10.0)	2 (11.8)	1.000
Stroke type			
LAA (%)	11 (27.5)	4 (23.5)	1.000
CE (%)	3 (7.5)	1 (5.9)	1.000
SVO (%)	25 (62.5)	11 (64.7)	1.000
SUE (%)	1 (2.5)	1 (5.9)	0.511
Life style			
Smoking (%)	18 (45.0)	7 (41.2)	1.000
Alcohol (%)	19 (47.5)	5 (29.4)	0.251

BMI, Body Mass Index; LAA, large artery arteriosclerosis; CE, cardiogenic embolism; SVO, small vessel occlusion; SUE, stroke of undetermined etiology.

**Table 6:** Comparisons of MI score and SSS score between the correspondence group and the noncorrespondence group.

	Correspondence group (*n* = 40)	Noncorrespondence group (*n* = 17)	*p* value
Visit 1 MI score	54.8 ± 25.7	45.9 ± 31.7	0.382
ΔMI score	14.3 ± 11.3	6.3 ± 9.3	0.003^*∗*^
Visit 1 SSS score	39.9 ± 10.4	38.1 ± 11.8	0.662
ΔSSS score	6.9 ± 4.0	3.5 ± 3.2	0.001^*∗*^

MI, Motricity Index; SSS, Scandinavian Stroke Scale.

^*∗*^ *p* < 0.05.

**Table 7:** Changes of the pattern probabilities in the correspondence group and the noncorrespondence group before and after the treatment.

	Before	After	*p* value
Correspondence group (*n* = 40)			
Probability of fire-heat pattern	62.6 ± 39.3^†^	51.1 ± 40.4	0.013^*∗*^
Probability of Yin Deficiency Pattern	9.8 ± 27.5	5.3 ± 17.9	0.337
Probability of Phlegm Dampness Pattern	6.2 ± 17.8	8.8 ± 16.9	0.149
Probability of Qi Deficiency Pattern	2.0 ± 10.3	3.6 ± 11.1	0.432
Noncorrespondence group (*n* = 17)			
Probability of fire-heat pattern	49.5 ± 37.0^†^	43.8 ± 35.7	0.374
Probability of Yin Deficiency Pattern	23.9 ± 40.1	32.5 ± 45.4	0.646
Probability of Phlegm Dampness Pattern	22.0 ± 36.7	12.8 ± 31.3	0.182
Probability of Qi Deficiency Pattern	0.0 ± 0.1	11.0 ± 26.0	0.050

^*∗*^ *p* < 0.05.

^†^ *p* < 0.05, compared with the probability of other patterns in the same group.
